# Ketamine induced synaptic plasticity operates independently of long-term potentiation

**DOI:** 10.1038/s41386-024-01895-2

**Published:** 2024-06-19

**Authors:** Michelle K. Piazza, Ege T. Kavalali, Lisa M. Monteggia

**Affiliations:** 1 Vanderbilt Brain Institute, Vanderbilt University, Nashville, TN 37240-7933 USA; 2 Department of Pharmacology, Vanderbilt University, Nashville, TN 37240-7933 USA

**Keywords:** Cellular neuroscience, Synaptic plasticity

## Abstract

Synaptic plasticity occurs via multiple mechanisms to regulate synaptic efficacy. Homeostatic and Hebbian plasticity are two such mechanisms by which neuronal synapses can be altered. Although these two processes are mechanistically distinct, they converge on downstream regulation of AMPA receptor activity to modify glutamatergic neurotransmission. However, much remains to be explored regarding how these two prominent forms of plasticity interact. Ketamine, a rapidly acting antidepressant, increases glutamatergic transmission via pharmacologically-induced homeostatic plasticity. Here, we demonstrate that Hebbian plasticity mechanisms are still intact in synapses that have undergone homeostatic scaling by ketamine after either systemic injection or perfusion onto hippocampal brain slices. We also investigated this relationship in the context of stress induced by chronic exposure to corticosterone (CORT) to better model the circumstances under which ketamine may be used as an antidepressant. We found that CORT induced an anhedonia-like behavioral phenotype in mice but did not impair long-term potentiation (LTP) induction. Furthermore, corticosterone exposure does not impact the intersection of homeostatic and Hebbian plasticity mechanisms, as synapses from CORT-exposed mice also demonstrated intact ketamine-induced plasticity and LTP in succession. These results provide a mechanistic explanation for how ketamine used for the treatment of depression does not impair the integrity of learning and memory processes encoded by mechanisms such as LTP.

## Introduction

Ketamine, an NMDA receptor antagonist, has attracted a great deal of attention for its rapid antidepressant action, which can be recapitulated in pre-clinical mouse experiments [1, 2]. At the molecular level, ketamine binds within the pore of spontaneously active, also referred to as “resting”, NMDA receptors to reduce calcium (Ca^2+^) influx into the postsynaptic neuron. This reduction of resting Ca^2+^ signaling alters intracellular signaling to ultimately increase local brain-derived neurotrophic factor protein (BDNF) synthesis, thereby strengthening excitatory neurotransmission and alleviating depressive phenotypes [3, 4]. In this way, ketamine’s inhibition of spontaneous NMDA receptor-mediated neurotransmission results in increased excitatory signaling and represents an example of pharmacologically-elicited homeostatic synaptic upscaling of plasticity.

Global regulation of synaptic strength via homeostatic plasticity contrasts with classical Hebbian plasticity, which is associative and input-specific in a positive feedback manner. In Hebbian-style plasticity, concurrent presynaptic and postsynaptic neuronal activation increases the strength of the connection only between the involved neurons [5–7]. An example of Hebbian plasticity is long-term potentiation (LTP), which is believed to be the synaptic correlate of learning and memory. Though distinct in underlying molecular signatures and time scales [8, 9], homeostatic and Hebbian plasticity have mechanistic overlaps, such as NMDA and AMPA receptor activity modulation. For example, homeostatic plasticity induced by ketamine is elicited by a decrease in NMDA-mediated signaling [3, 4], while LTP requires an increase in glutamatergic activity at the NMDA receptor [10, 11]. Despite this difference, both mechanisms lead to an upregulation of AMPA-mediated activity, albeit by differing signaling pathway involvement [9, 12], that results in an overall increase in excitatory neurotransmission [3, 4, 10, 11]. The multiplicative nature of homeostatic plasticity is thought to preserve synaptic weights structured by Hebbian plasticity mechanisms, such as LTP, and may help stabilize neural circuits to prevent oversaturation of Hebbian plasticity [13–15]. However, much remains to be learned regarding the interaction of homeostatic and Hebbian plasticity at the synaptic level.

Here, we investigated whether ketamine-induced homeostatic synaptic plasticity affects the ability of hippocampal synapses to undergo LTP induction utilizing both systemically administered and, separately, directly perfused low-dose ketamine. Additionally, we considered the effect of stress exposure on this relationship to recapitulate a depression-like milieu of ketamine action. We observed that ketamine’s impact on synaptic activity does not impair subsequent LTP in either administration paradigm, providing a synaptic basis to the clinical findings that ketamine’s antidepressant action does not impact learning and memory performance.

## Materials and methods

### Animals

Adult male and female mice from Jackson Labs were kept on a 12 h light/dark cycle at ambient temperature (23 ± 3 °C) and humidity (50 ± 20%) with access to food and water *ad libitum*. All animal procedures were performed in accordance with the guidance for the care and use of laboratory animals and were approved by the institutional animal care and use committee at Vanderbilt University.

### Stress Induction

Male and female mice were provided *ad libitum* access to either 100 μg/mL corticosterone (CORT) or 1% ethanol as the vehicle control (CTL), both diluted in tap water, for 14 days. Each cage was provided with two identical bottles of drinking water which were refilled every 48 h to ensure drug potency.

### Sucrose preference testing

After 14 days of CORT or CTL exposure, all mice were single-housed in clean cages and deprived of water overnight. On the following day, each mouse was given one bottle of tap water and one bottle of 2% sucrose solution for a total of 3 days. The position of the bottles was switched on the second day of exposure to prevent place preference. All bottles were weighed before and after testing to measure consumption. Sucrose preference was defined as the amount of sucrose solution consumed divided by the total amount of liquid consumed for each mouse. Sucrose preference under 75% was considered an anhedonia-like phenotype indicative of stress induction consistent with literature in the field [16]. Mice were excluded from further testing if they were exposed to CORT and preferred sucrose over 75%, or if they were in the control group and preferred sucrose at less than 75%. In total, 12 out of 46 animals were excluded, and animal dropout was consistent between groups (three male control, three male CORT, four female control, two female CORT).

### Hippocampal slice electrophysiology

**Fig. 1:**
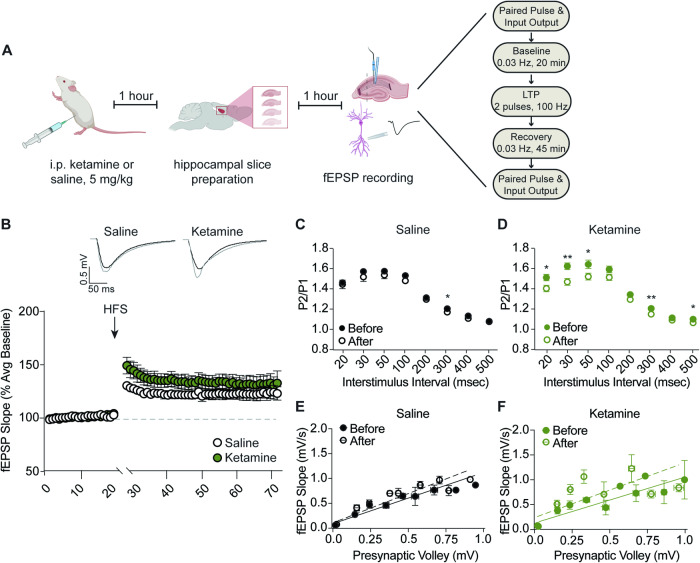
Systemic ketamine does not affect LTP magnitude in hippocampal slices. **A** Schematic depiction of experimental procedure and protocol for fEPSP measurement of LTP in the Schaffer collateral pathway of the hippocampus (CA3 to CA1). **B** Systemic ketamine treatment (5 mg/kg, i.p.) caused a slight but not significant increase in LTP magnitude induced by high-frequency stimulation relative to saline treated (5 mL/kg, i.p.) controls. (Saline *n* = 6; Ketamine *n* = 8; two-way ANOVA effect of treatment F_(1,13)_ = 2.069, *p* = 0.1740). **C** LTP induced a decrease in paired-pulse ratio in saline-treated mice at 300 ms interstimulus interval. (multiple unpaired *t*-tests). **D** Ketamine-treated mice demonstrated a reduction in paired-pulse ratio across a range of interstimulus intervals. (multiple unpaired *t*-tests). **E**, **F** The relationship of presynaptic input intensity to resulting postsynaptic response was slightly increased by LTP in saline and ketamine-treated mice. (Linear regression; saline before slope = 1.070, saline after slope = 1.220, *p* = 0.2377; ketamine before slope = 0.9599, ketamine after slope = 1.147, *p* = 0.3866). *p < 0.05; **p < 0.01.

Adult male and female mice were anesthetized with isofluorane and decapitated. Brains were removed and immersed in ice-cold dissection buffer containing the following: 2.6 mM KCl, 1.25 mM NaH_2_PO_4_, 26 mM NaHCO_3_, 0.5 mM CaCl_2_, 5 mM MgCl_2_, 212 mM sucrose, and 10 mM glucose for 4–6 min for tissue dissection. Hippocampi were collected and cut with a vibratome into 400 μm-thick transverse sections in ice-cold dissection buffer continuously aerated with 95% O_2_/5% CO_2_. Area CA3 was surgically removed immediately after sectioning to prevent recurrent cellular activity during recording. Sections were allowed to recover for 3 h at 30 °C in oxygenated artificial cerebrospinal fluid (ACSF) containing the following: 124 mM NaCl, 5 mM KCl, 1.25 mM NaH_2_PO_4_, 26 mM NaHCO_3_, 2 mM CaCl_2_, 2 mM MgCl_2_ and 10 mM glucose, pH 7.4, and continuously aerated with 95% O_2_/ 5% CO_2_. Slice recovery was shortened to 1 h for investigation of the effects of systemically delivered ketamine (Fig. 1) to ensure that synaptic phenotypes were examined in the time window of ketamine’s acute antidepressant effects. Following recovery, hippocampal slices were transferred to the recording chamber and continuously perfused with oxygenated ACSF at a rate of 2–3 mL/min at 30 ± 0.5 °C. Field Excitatory Postsynaptic Potentials (fEPSPs) were evoked by inserting a concentric bipolar stimulating electrode (FHC) and an extracellular recording electrode filled with ACSF (resistance 1-2 MΩ) in the CA1 *stratum radiatum* proximally below molecular level.

In all experiments, paired-pulse facilitation was elicited by paired stimulations at decreasing interstimulus intervals (ISIs) of 500, 400, 300, 200, 100, 50, 30, and 20 ms at the stimulus intensity inducing 60% of the slice’s maximal response. The fEPSP slope of pulse 2 (P2) was divided by pulse 1 (P1) to give a ratio representing presynaptic release probability. Paired pulse stimulation was applied before baseline and following recovery after LTP. The efficacy of the post-synaptic response was measured by delivering stimuli at increasing intensity in a stepwise fashion from 0 to the intensity producing the maximum response for each slice. The magnitude of the presynaptic fiber volley peak was plotted against the resulting fEPSP slope to measure postsynaptic strength. Similar to paired-pulse facilitation, input/output was measured before baseline and following LTP recovery.

After paired-pulse facilitation and input/output, baseline responses were collected every 30 s (0.03 Hz) using an input stimulus intensity that induced 60% of the slice’s maximum response. In line with previous work from our lab, baseline recordings were measured for 20 min or until stable. In the ketamine groups, baseline was followed by 30 min of drug perfusion in the absence of stimulation. One single response was collected to assess the immediate effect of drug perfusion, and stimulation was stopped to allow drug wash out at rest for 60 min. Hippocampal slices were then stimulated again at 0.03 Hz for 20 min to assess the sustained effect of drug perfusion. Following this recovery period, or following baseline for the groups that did not receive ketamine, LTP was induced by high-frequency stimulation (HFS). Two pulses of 100 Hz for 1 s each were delivered with a 5 min waiting period after each. Stimulation was then resumed at 0.03 Hz for 45 min to assess LTP induction efficacy.

Experimental time courses for electrophysiology data were prepared by normalizing the fEPSP response at each stimulation to that recording’s average baseline. Responses were then averaged within treatment groups. Time courses were binned in 1 min intervals for visualization only. Data from male and female mice were combined due to a lack of observed differences in basal synaptic function. Recordings with unstable responses were excluded from analysis.

### Drugs

Ketamine (100 mg/mL, Hospira) was diluted in saline (0.9% NaCl, Intermountain Life Sciences) to 1.0 mg/mL and injected intraperitoneally (i.p.) at 5 mg/kg. Saline was injected i.p. at 5 mL/kg as a vehicle control with similar treatment volume. CORT, (Sigma) was kept at a stock concentration of 10 mg/mL in EtOH and then diluted in water to 100 μg/mL. Ethanol (EtOH, 200 proof, Danco Labs, Inc.) was diluted in water to a 1% solution. Sucrose (Sigma) was dissolved in water to a 2% concentration. Tap water was used for all animal drinking solutions for consistency with their normal water supply. For electrophysiology experiments, ketamine (20 μM, Hospira) was prepared in ACSF and administered via syringe pump perfusion at a flow rate of 18 mL/h.

### Statistical analysis

All bar graph data are presented as mean ± SEM and analyzed with an unpaired t-test. Field recording responses were analyzed with a two-way ANOVA accounting for treatment group and time. Paired pulse data were analyzed with multiple t-tests at each interstimulus interval. Input/output data were fit with a linear regression and slopes were compared. Data visualization and statistical analysis were done using GraphPad Prism (versions 8-10).

## Results

### Systemic ketamine does not affect LTP magnitude in hippocampal slices

We first assessed the impact of systemically delivered ketamine on subsequent LTP induction in hippocampal slices prepared from treated mice. Male mice were administered ketamine (5 mg/kg, i.p.) or saline control (0.9% saline, 5 mL/kg, i.p.) and sacrificed for hippocampal slice preparation 1 h later. Slices were then allowed to recover for 1 h before recording of fEPSPs in the Schaffer collateral pathway from the CA3 to CA1 subregions of the hippocampus (Fig. 1A). After a stable baseline was established, slices received tetanic stimulation (HFS, two 100 Hz pulses for 1 s each, with a 5 min rest after each) followed by a 45 min recovery period at baseline stimulation frequency. We tested multiple HFS stimulation protocols for LTP induction in accordance with previous work in the field employing one to four 100 Hz pulses. We chose to use two pulses of 100 Hz because this stimulation protocol induced strong and stable LTP in male and female mice. Paired pulse facilitation and input/output were measured before baseline and after LTP recovery (Fig. 1A).

Systemic ketamine treatment had little impact on the magnitude of LTP induced by HFS compared to saline treated controls, inducing a small but not significant increase in LTP strength (Fig. 1B). Surprisingly, LTP caused a decrease in paired-pulse stimulation at an interstimulus interval (ISI) of 300 ms in saline-treated mice, and across a range of ISIs in ketamine treated mice (Fig. 1C and D). Mice of both treatment groups also demonstrated a modest leftward shift in the relationship between presynaptic input intensity and resulting fEPSP slope, indicating an increase in postsynaptic efficacy, but these effects failed to reach statistical significance (Fig. 1E and F). Collectively, these results demonstrate that systemically administered low-dose ketamine treatment does not impair LTP.

### CORT exposure induces a behavioral stress phenotype in mice

**Fig. 2:**
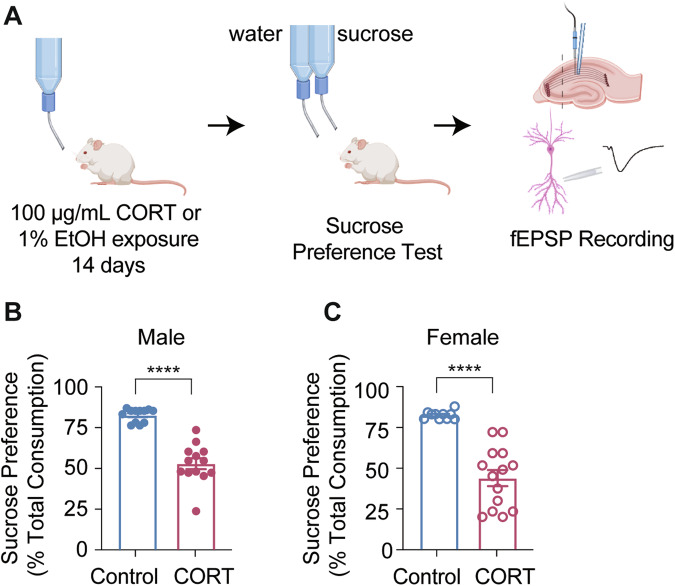
CORT exposure induces a behavioral stress phenotype in mice. **A** Schematic depiction of the experimental protocol. **B** Male mice exposed to CORT showed a reduction in sucrose consumption relative to control, indicating a stress-like phenotype. (Control *n* = 12; CORT *n* = 13; unpaired *t*-test, *p* < 0.0001). **C** Female mice exposed to CORT showed a reduction in sucrose consumption relative to control, indicating a stress-like phenotype. (Control *n* = 10; CORT *n* = 14; unpaired *t*-test, *p* < 0.0001). ****p < 0.0001.

While systemic ketamine administration seems to have little effect on LTP induction, this experimental paradigm presents the inevitable possibility that procedures that intervene between systemic in vivo injection and ex-vivo slice recordings may interfere with the interaction of ketamine-induced synaptic plasticity and LTP. Therefore, next, we sought to test this premise more directly using perfusion of ketamine onto hippocampal brain slices. We also utilized a CORT exposure model of stress induction in both male and female mice to better recapitulate the clinical circumstances under which ketamine may be used as an antidepressant. Mice were given *ad libitum* access to either 100 μg/mL CORT or 1% ethanol, the vehicle control (CTL), in their drinking water for 14 days. All mice then underwent sucrose preference testing to confirm a stress phenotype. Mice that were exposed to CORT and demonstrated a sucrose preference of less than 75%, and control mice that demonstrated a sucrose preference of greater than 75% were used for investigation of hippocampal neurotransmission via field electrophysiology (Fig. 2A). We verified that CORT exposure reduced sucrose preference (indicative of a stress phenotype) in both male and female mice (Fig. 2B and C). Mice that underwent CORT treatment demonstrated small increases in paired-pulse ratio at select low ISIs but did not show a change in postsynaptic efficacy or baseline fEPSP responses (data not shown). Taken together, these data suggest that CORT exposure effectively induced a behavioral stress phenotype in mice, but had little impact on the overall strength of synaptic activity.

### Basal synaptic function is comparable between male and female mice

**Fig. 3:**
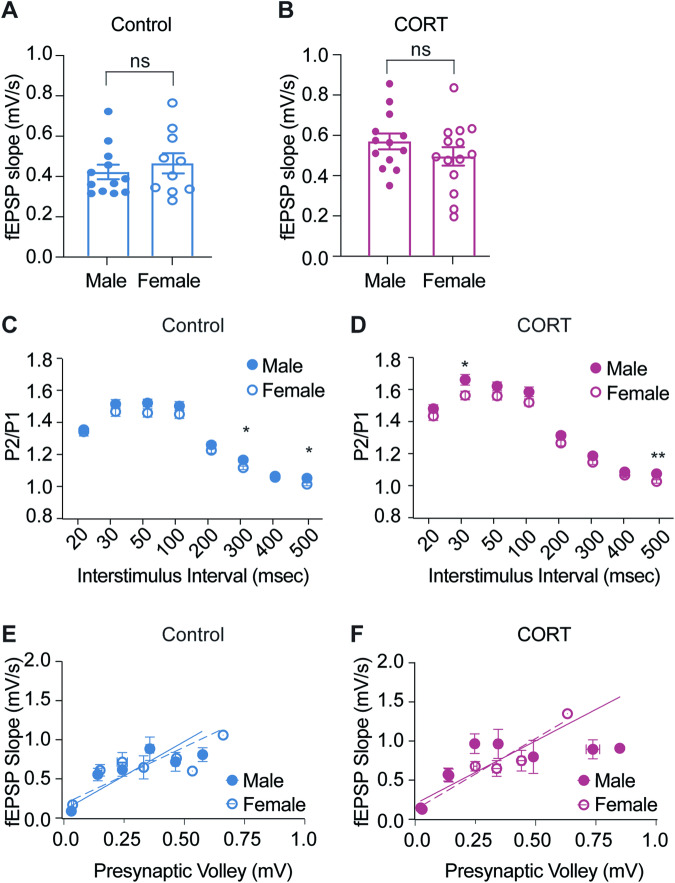
Basal synaptic function is comparable between male and female mice. **A**, **B** Average baseline fEPSP slopes in the Schaffer collateral pathway of the hippocampus (CA3 to CA1) did not differ between male and female mice exposed to control or CORT treatment. (Male Control *n* = 12, Female control *n* = 10, unpaired *t*-test *p* = 0.4872; Male CORT *n* = 13; Female CORT *n* = 14; unpaired *t*-test *p* = 0.2334). **C** Females in the control treatment group showed a lower paired-pulse ratio than males only at interstimulus intervals of 300 and 500 ms. (multiple unpaired *t*-tests). **D** CORT-treated female mice showed a reduction in paired-pulse ratio at interstimulus intervals of 30 and 500 ms compared to CORT-treated males. (multiple unpaired *t*-tests). **E** Post synaptic strength did not differ between control-treated male and female mice. (Linear regression; Male slope = 1.791; Female slope = 1.481; *p* = 0.2848). **F** Sex did not alter postsynaptic strength in CORT-treated mice. (Linear regression; Male slope = 1.640; Female slope = 1.854; *p* = 0.4511). *p < 0.05; **p < 0.01.

The influence of sex on basal synaptic function was examined using paired-pulse ratio and input/output data collected from these CTL and CORT-treated mice prior to ketamine treatment and/or LTP induction. Baseline fEPSP strength was comparable between males and females in both treatment groups (Fig. 3A and B). When we examined presynaptic function, we found that females exhibit a lower paired-pulse ratio than males only at ISIs of 300 and 500 ms in the control group, and 30 and 500 ms in the CORT group (Fig. 3C and D). These changes may indicate an increase in presynaptic release probability. However, postsynaptic efficacy was comparable across sexes in both treatment conditions (Fig. 3E and F). The lack of sex or stress treatment effect on baseline fEPSP strength or postsynaptic efficacy suggests that sex differences in paired-pulse facilitation may have little influence on overall synaptic function.

### CORT exposure does not impair LTP induction

**Fig. 4:**
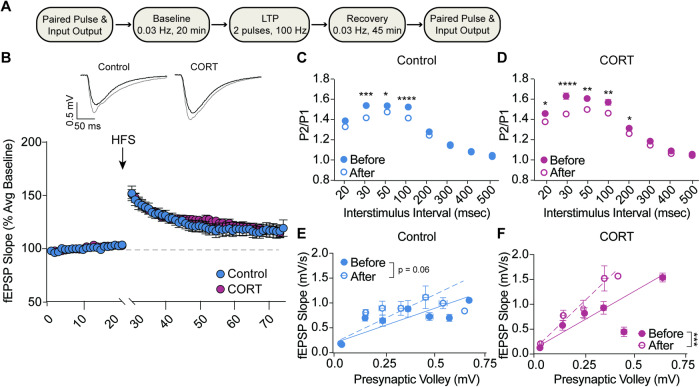
CORT exposure did not impair LTP induction. **A** Schematic depicting experimental protocol for fEPSP measurement of LTP in the Schaffer collateral pathway of the hippocampus (CA3 to CA1). **B** LTP induced by high-frequency stimulation was not impaired by CORT exposure. (Control *n* = 12, CORT *n* = 15; two-way ANOVA effect of stress F_(1,25)_ = 0.1871, *p* = 0.6691). **C**, **D** LTP induction reduced paired-pulse ratio in both control and CORT-treated mice, possibly indicating an increase in presynaptic release probability across a range of low interstimulus intervals. (multiple unpaired *t*-tests). **E**, **F** Postsynaptic strength was increased in control and CORT-treated mice, indicated by a leftward shift in the input/output relationship slope. (Linear regression; Control slope before LTP = 1.450, control slope after LTP = 2.004, *p* = 0.06; CORT slope before LTP = 2.407, CORT slope after LTP = 4.022, *p* < 0.001). *p < 0.05; **p < 0.01; ***p < 0.001; ****p < 0.0001.

We next examined how stress exposure via CORT drinking water influences classic Hebbian plasticity. Given the lack of robust sex differences in basal synaptic function, we combined electrophysiology data from male and female mice. Hippocampal brain slices were prepared for measurement of fEPSPs in the Schaffer collateral pathway, which were measured following the same timeline as used in Fig. 1; after a stable baseline was achieved, LTP was induced by HFS followed by a 45 min recording period at baseline stimulation frequency (0.03 Hz). Paired pulse facilitation and input/output were measured before baseline and after LTP recovery (Fig. 4A). We found that stress exposure did not alter either the induction magnitude or the maintenance strength of LTP in male or female mice (Fig. 4B). Unexpectedly, LTP induction was associated with a small but significant decrease in paired-pulse facilitation across a range of ISIs in both control and CORT exposed mice (Fig. 4C and D). Mice of both treatment groups also demonstrated a leftward shift in the relationship of presynaptic fiber volley to resulting fEPSP slope (Fig. 4E and F). These data indicate that LTP induction by tetanic HFS increases postsynaptic strength and may, to some extent, alter presynaptic release probability in a manner that is not affected by prior exposure to CORT.

### Ketamine-induced synaptic plasticity does not occlude subsequent LTP

**Fig. 5:**
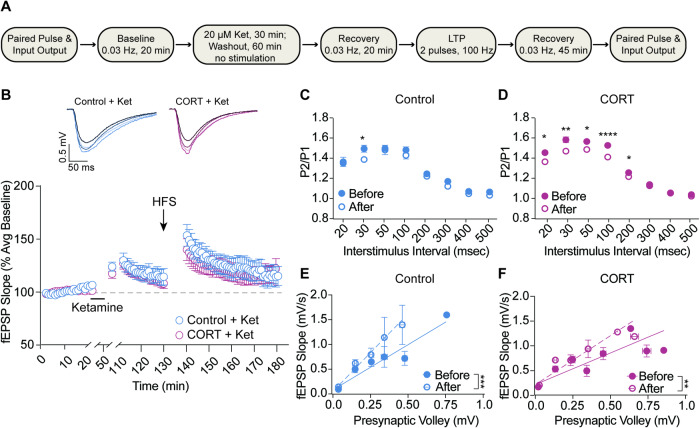
Ketamine-induced synaptic plasticity did not occlude subsequent LTP. **A** Schematic depicting experimental protocol for fEPSP measurement of the effects of ketamine and LTP in the Schaffer collateral pathway of the hippocampus (CA3 to CA1). **B** Hippocampal brain slices showed an increase in the strength of excitatory neurotransmission following ketamine perfusion (20 μM). This homeostatic plasticity did not occlude subsequent LTP induction, as excitatory synaptic strength was further increased following high-frequency electrical stimulation to induce LTP. Example traces denote average response during baseline, ketamine recovery, early LTP (minutes 140–145), and full LTP recovery (minutes 140–185) from darkest to lightest shade, respectively. (Control + Ket *n* = 10; CORT + Ket *n* = 12; two-way ANOVA for ketamine induced plasticity stress effect F_(1,20)_ = 0.1523, *p* = 0.7005; two-way ANOVA for LTP stress effect F_(1,20)_ = 0.1843, *p* = 0.6723). **C**, **D** Paired pulse facilitation was decreased after ketamine perfusion and LTP induction in control mice at a 30 ms interstimulus interval, and in CORT-treated mice at a range of low interstimulus intervals. (multiple unpaired *t*-tests). **E**, **F** Postsynaptic efficacy was strengthened following ketamine perfusion and LTP induction in both CORT and control exposed mice. (Control + ket slope before LTP = 1.893, control + ket slope after LTP = 3.253, *p* < 0.001; CORT + ket slope before LTP = 1.324, CORT + ket slope after LTP = 2.053, *p* < 0.01). *p < 0.05; **p < 0.01; ****p < 0.0001.

After examining the isolated effect of CORT treatment on LTP, we investigated the intersection of homeostatic and Hebbian plasticity mechanisms using ketamine, a known pharmacological inducer of homeostatic plasticity [3, 4, 17]. Hippocampal responses were measured as before, but in these experiments, slices also received acute perfusion of ketamine prior to LTP induction. Following establishment of a stable baseline response, ketamine was perfused for 30 min in the absence of electrical stimulation (Fig. 5A). Immediately following ketamine application, fEPSP strength was increased in the hippocampus of both control and CORT-treated mice. In line with previous evidence, this synaptic potentiation persisted following 1 h of drug washout at rest. The magnitude of ketamine-induced potentiation was similar across groups, suggesting that stress does not impact ketamine’s efficacy. We then delivered HFS as before to produce LTP. Interestingly, LTP induction remained intact in the hippocampus of both control and CORT-exposed mice previously treated with ketamine (Fig. 5B). These data show ketamine-mediated synaptic potentiation is mechanistically distinct from Hebbian LTP, and that synapses are able to undergo both types of plasticity in succession.

To uncover the presynaptic and postsynaptic contributions on the intersection of homeostatic and Hebbian plasticity, we assessed paired pulse ratio and input/output slope. We found that combined ketamine and LTP decreased paired-pulse ratio at an interstimulus interval of 30 ms in control, and at a range of low ISIs in CORT-treated hippocampal slices (Fig. 5C and D), each suggesting a possible increase in presynaptic release probability. Furthermore, both control and CORT mice demonstrated a strong leftward shift in the slope of the input/output curve, indicating strengthening of the postsynaptic response (Fig. 5E and F).

## Discussion

Neuronal synapses exist in a dynamic state: they can undergo changes in strength, known as synaptic plasticity, by numerous mechanistically distinct processes [4, 9, 17–19]. Homeostatic plasticity is a well-established form of synaptic plasticity whereby changes in neuronal activity lead to a global increase or decrease in the strength of all of a neuron’s synaptic inputs in a negative feedback loop [13]. In this way, after a perturbation of the system, homeostatic plasticity restores a neuron to its original state of activity and prevents saturation of activity levels in either the positive or negative direction. Our earlier studies have shown that ketamine acts via blocking resting NMDA receptor-mediated neurotransmission and elicits a subsequent increase in excitatory signaling to re-establish a homeostatic level of neurotransmission via a process known as synaptic scaling [13, 17, 20].

In this study, we found that ketamine’s effects on excitatory hippocampal synapses following systemic injection or bath perfusion did not occlude subsequent induction or maintenance of classic LTP. Systemic administration of 5 mg/kg ketamine caused a small but not significant increase in LTP. This finding is consistent with previous work demonstrating that systemically delivered ketamine, at slightly higher doses than used in this study, increased or did not influence LTP magnitude over a range of treatment durations from 30 min to 72 h [21–26]. Furthermore, we used chronic exposure to CORT in mice to model a stress phenotype and observed that ketamine-induced synaptic potentiation via bath application also did not alter the ability to induce LTP in this type of stress model. These data show that the antidepressant action of ketamine, which strengthens hippocampal synapses via a form of homeostatic plasticity, is distinct from LTP, a form of Hebbian plasticity. This finding provides a synaptic basis for the clinical observation that ketamine’s antidepressant action does not impair learning and memory.

The mechanistic distinctions demonstrated here add to a growing body of literature indicating that homeostatic and Hebbian plasticity are discrete molecular processes that can exist in synchrony [9, 12, 15, 27, 28]. The use of ketamine to induce synaptic scaling in this study provides an important contrast to previous work by demonstrating that the various pharmacological methods that have shown efficacy in provoking homeostatic plasticity may operate via divergent cellular machinery. For example, we show that bath-applied ketamine caused homeostatic synaptic potentiation that did not interfere with subsequent LTP. However, synaptic upscaling by acute retinoic acid treatment increased AMPA receptor-mediated activity and impaired LTP induction [9], whereas tetrodotoxin (TTX) induced scaling increased both AMPA and NMDA expression and actually enhanced LTP [27, 28]. Taken together, these studies demonstrate that homeostatic plasticity induction methods are not unequivocal, and they must be considered when studying the interaction between homeostatic and Hebbian plasticity.

Ketamine’s effects on synaptic physiology and behavior are highly influenced by drug concentration. The ability of low-dose ketamine to augment fEPSPs at CA3-CA1 synapses is well documented [3, 4, 29–31], and is mediated postsynaptically at CA3-CA1 synapses similar to ketamine’s antidepressant-like behavioral effects [4, 31]. At higher doses, ketamine does not trigger synaptic potentiation or antidepressant-like effects but rather produces increased locomotor activity and impairments in learning and memory [30, 32]. These high concentrations of ketamine reflect the dose ranges encompassing illicit recreational use to anesthetic use in humans, and are used to model aspects of schizophrenia-related behavior in mice [32, 33]. Conversely, low-dose ketamine associated with hippocampal synaptic potentiation and antidepressant-like behavioral effects do not alter locomotor activity or impact learning and memory [3, 4, 29–31, 34, 35]. This dichotomy between the dose of ketamine and the behavioral and synaptic effects provides a rationale for why low-dose administration of ketamine has been a key factor in its clinical efficacy countering depression.

LTP is known to increase excitatory neurotransmission via a postsynaptic mechanism, although a variety of both pre and postsynaptic mechanisms have been described since its discovery [11, 36, 37]. The postsynaptic requirement for ketamine-induced synaptic potentiation, as well as for LTP, suggests potential segregation in synaptic signaling such that they can co-exist without interfering with one another [9, 12]. In the current study, rather unexpectedly after LTP stimulation, we observed a decrease in paired-pulse facilitation in control and CORT-treated mice of both sexes that accompanied the increase in postsynaptic strength. Importantly, the effects of electrical stimulation, as to induce LTP, can be stored in synaptic connections such that short-term plasticity is evident when measured immediately after stimulation, and even after a delay of up to 6 h. Strikingly, the degree of lasting LTP is equivalent even after such a delay in resuming stimulation [38]. This short-term potentiation (STP) is considered to be expressed presynaptically, as the decay of STP into LTP is modulated by the probability of neurotransmitter release [39]. Furthermore, HFS of hippocampal neurons can increase spontaneous neurotransmission for up to 30 min, demonstrating another way in which electrical stimulation can induce enduring effects on presynaptic function [40]. Our observation that paired-pulse facilitation was decreased, which may indicate an increase in presynaptic release probability, 45 min after tetanic stimulation may thus reflect a lasting consequence of tetanic stimulation at CA3-CA1 synapses via stored short-term plasticity, suggesting that both pre and postsynaptic mechanisms may contribute to long-lasting plasticity at hippocampal synapses.

We used chronic CORT administration via water as a proxy to model stress and examine the impact on synaptic plasticity processes. In our model, chronic CORT was sufficient to induce anhedonia, although it did not impair LTP. Some stress studies, depending on the type of stressor and duration, have reported a reduction in LTP strength [22, 41–47], although this is not unequivocal. LTP deficits appear to be more pronounced in models of more severe stressors, such as social defeat or disturbance [41, 42, 47, 48], restraint [49–51], footshock [49, 52], and other behavioral paradigms [43, 44, 48, 53, 54]. Systemic administration of CORT has also been reported to induce LTP deficits [45–48, 55], suggesting this physically stressful experience may compound the effect of CORT alone. In support of this notion, milder behavioral stressors that are sufficient to increase CORT levels are not always associated with LTP deficits [43, 52, 56–58]. Thus, while particular stressors can cause deficits in LTP, these LTP deficits do not always accompany stress exposure-induced changes in anhedonia behavior.

We observed that ketamine was equally efficacious in modulating synaptic function in naïve and chronic CORT-treated mice. Indeed, previous work from our lab and others shows that ketamine’s behavioral antidepressant-like effect is evident even in the absence of stress induction in rodents [3, 4, 35]. Additionally, we showed that low-dose ketamine augmented fEPSPs at CA3-CA1 synapses and did not interfere with the induction of LTP in naïve or chronic CORT-treated male or female mice. The lack of observed sex differences is consistent with previous pre-clinical work [31, 59] as well as clinical data showing that ketamine produces antidepressant action similarly in male and female patients [60].

Synaptic plasticity is a crucial neuronal function that is not homogenous. Various types of synaptic plasticity exist, and the mechanisms by which they occur and how they interact remain to be elucidated. Here, we demonstrated that ketamine-induced upscaling of homeostatic plasticity did not impair subsequent induction of classic Hebbian plasticity by LTP when administered systemically or by direct perfusion onto hippocampal slices. Importantly, neither the efficacy of ketamine in inducing this plasticity, the overall magnitude of LTP, nor the interaction between the two was impacted by stress via CORT exposure in drinking water. Future work will be important to tease apart the mechanistic interplays between synaptic plasticity and stress and the nuances of the induction methodology of each. Taken together, the findings presented here provide a mechanistic understanding of why learning and memory, considered to be encoded by LTP, are not impaired by ketamine’s modulation of synaptic activity.

## Supplementary information

Data Availability

## Data Availability

The datasets from this paper are presented in the supporting file.
